# Rubus: A compiler for seamless and extensible parallelism

**DOI:** 10.1371/journal.pone.0188721

**Published:** 2017-12-06

**Authors:** Muhammad Adnan, Faisal Aslam, Zubair Nawaz, Syed Mansoor Sarwar

**Affiliations:** Punjab University College of Information Technology, University of the Punjab, Lahore, Pakistan; Wroclaw University of Technology, POLAND

## Abstract

Nowadays, a typical processor may have multiple processing cores on a single chip. Furthermore, a special purpose processing unit called Graphic Processing Unit (GPU), originally designed for 2D/3D games, is now available for general purpose use in computers and mobile devices. However, the traditional programming languages which were designed to work with machines having single core CPUs, cannot utilize the parallelism available on multi-core processors efficiently. Therefore, to exploit the extraordinary processing power of multi-core processors, researchers are working on new tools and techniques to facilitate parallel programming. To this end, languages like CUDA and OpenCL have been introduced, which can be used to write code with parallelism. The main shortcoming of these languages is that programmer needs to specify all the complex details manually in order to parallelize the code across multiple cores. Therefore, the code written in these languages is difficult to understand, debug and maintain. Furthermore, to parallelize legacy code can require rewriting a significant portion of code in CUDA or OpenCL, which can consume significant time and resources. Thus, the amount of parallelism achieved is proportional to the skills of the programmer and the time spent in code optimizations. This paper proposes a new open source compiler, Rubus, to achieve seamless parallelism. The Rubus compiler relieves the programmer from manually specifying the low-level details. It analyses and transforms a sequential program into a parallel program automatically, without any user intervention. This achieves massive speedup and better utilization of the underlying hardware without a programmer’s expertise in parallel programming. For five different benchmarks, on average a speedup of 34.54 times has been achieved by Rubus as compared to Java on a basic GPU having only 96 cores. Whereas, for a matrix multiplication benchmark the average execution speedup of 84 times has been achieved by Rubus on the same GPU. Moreover, Rubus achieves this performance without drastically increasing the memory footprint of a program.

## 1 Introduction

Since the beginning of computing, computer users desire to have higher computing speed to perform tasks previously considered near impossible. A few examples of such tasks are realistic weather forecast, disaster predictions, calculating moves of complex games, and real-time simulations. Historically, efforts have been made in digital computing to increase processing speed mainly using three techniques: 1) increasing the clock speed of processor, 2) decreasing memory access time, and 3) improving instruction level parallelism (ILP). These techniques resulted in significant increase in computing speed over the years, until they reached to their limits. That is, due to power wall, memory wall and ILP wall it has now become excessively difficult to further enhance the execution speed using aforementioned techniques [2].

Thus, since the start of 21st century, in order to excel in business and fulfill user’s computing needs, CPU vendors have been forced to move from single-core to multi-core architecture. Nowadays, a processor has several processing cores on a single chip. In addition, a special processing unit, called GPU, which was initially designed for 2D/3D games, is also made available in personal computers, laptops and mobile devices. A modern GPU may contain up to several thousands simple processing units optimized for graphic processing but may be also used for general purpose computing. However, traditional programming languages, which were designed to deal with single core machines, cannot fully utilize the multi-core CPUs and GPUs efficiently. To this end, a few low-level languages including Compute Unified Device Architecture (CUDA) [3] and Open Computing Language (OpenCL) have been introduced to exploit the parallelism capabilities of the underlying hardware. The main shortcoming of these languages is that the programmer needs to specify all the complex details about how to distribute the code on multiple cores for parallel execution. Thus, the amount of parallelism achieved is proportional to the skills of the programmer and the time spent on code optimization. Furthermore, the low-level code written in such languages becomes complex and difficult to understand, debug and maintain as it contains program logic as well as commands to distribute data across multiple cores and merge results.

There are some high level APIs like JCUDA [4], JOCL [5] and JavaCL [6] which provide Java bindings for these languages. Some other APIs including Aparapi [7] and Rootbeer [8] provide a high-level interface to work with GPUs and Multi-core CPUs. To use these APIs and bindings, one needs to learn and understand the parallelism techniques and the methodologies of actual GPU programming languages. However, parallel programming is not common as it is more difficult than sequential programming. Hence, either application programmers should be trained to always write parallel code or there should be an intermediate layer, a compiler, that may analyze the code, find out the portions of the code that may run in parallel and seamlessly transform them to run in parallel on multiple processing units. The option of having such a compiler is preferred as it does not require the programmer to have the knowledge of parallel programming techniques and it can transform the legacy code as well.

This paper presents Rubus [1], an open source compiler that can automatically transform a sequential program to a parallel program without requiring any input from the programmer. Thus, a programmer who has no knowledge of the underlying hardware or know-how of parallel programming can benefit from the hardware parallelism capabilities by simply compiling a sequential program using Rubus. Rubus relieves the burden of learning new languages, rewriting the code and specifying low-level details needed to parallelize the code. It aims to provide seamless data level parallelism by exploiting the massive computational power of GPUs and multi-core CPUs, without writing any extra code. The portion of the code that consumes the major part of total execution time usually consists of loops. A massive data level parallelism can be achieved by executing concurrent loop iterations in parallel. Rubus seamlessly finds out the loops and analyses them. In case, different iterations of the loops are independent or have a manageable dependency, Rubus transforms them into OpenCL and executes them in parallel. This achieves a massive boost in the program’s execution speed by utilizing the underlying parallel hardware efficiently. Instead of optimizing the source code, Rubus transforms Java bytecode directly, thus making Rubus a compiler of dozens of programming languages that generate Java bytecode.

According to our knowledge, there is no other work like Rubus in Java which could analyze code automatically and transform it into OpenCL to make it run in parallel on multi-core CPUs and GPUs. Java-GPU, on which Rubus is based, transforms code into CUDA that limits it to Nvidia’s GPUs only. In contrast, Rubus’s transformed code is compatible with the CPUs and GPUs from dozens of vendors. Furthermore, Java-GPU transforms code into C language and compiles it into an object (i.e.,.o) library that makes the code platform dependent. It also requires the C toolchain and CUDA toolkit to be configured on the machine. On the other hand, Rubus works purely in Java and generates platform independent and portable code.

The accuracy of the transformed code by Rubus is carefully maintained, that is, the output of a program is not affected during the analysis and optimization of the bytecode. The programs having loops and manageable dependencies between loop iterations execute in parallel and faster using Rubus. However, if there is a small set of operations on a big set of data, it may cause a big data transfer overhead from main memory to GPU’s memory and vice versa. Thus, Rubus is useful if a program has reasonably large computation requirements.

Following are the main contributions of Rubus.

1. Rubus provides massive parallelism and achieves execution speedup for different applications related to various domains of life.
2. Existing solutions require a programmer to write and optimize code in a specific way using language specific tools and techniques. Thus, they require the programmer to be trained first. In contrast, with Rubus, a programmer doesn’t need to modify or upgrade the code to achieve parallelism. Therefore, a programmer doesn’t require any extra training.
3. Rubus works with Java bytecode. Thus, it is compatible with any programming language that generates intermediate Java bytecode.
4. Rubus automatically selects the best available device (CPU/GPU) at runtime for the execution of transformed code. Currently, the device having the largest amount of computing units is considered the best. If a computer consists of more than one GPU units, the GPU having maximum computing units is selected.
5. Existing work mainly targets GPUs from a specific vendor, i.e., Nvidia. In contrast, Rubus works with different GPUs and CPUs from multiple vendors, e.g., Intel, Nvidia, AMD and Mali’s.
6. Rubus performs automatic analysis of the bytecode to identify the portion of the code that can be parallelized. Optionally, a programmer may insert some directives/annotations to make static analysis more accurate.
7. OpenCL has some limitations that make code transformation difficult. For example, OpenCL does not support the goto statement, which is a major part of Java bytecode. Conditional statements in Java source code generate goto statements in Java bytecode. Rubus avoids OpenCL limitations to some extent by reversing the conditions in order to support simple conditional statements within the loop body.

The paper is organized as follows. Section 2 describes the methodology used in the design of Rubus compiler. Section 3 describes the experiments conducted and discusses results. Section 4 discusses related works. Finally, Section 5 concludes the paper while we provide future work and limitations of Rubus in Section 6.

## 2 Methodology

**Fig 1:**
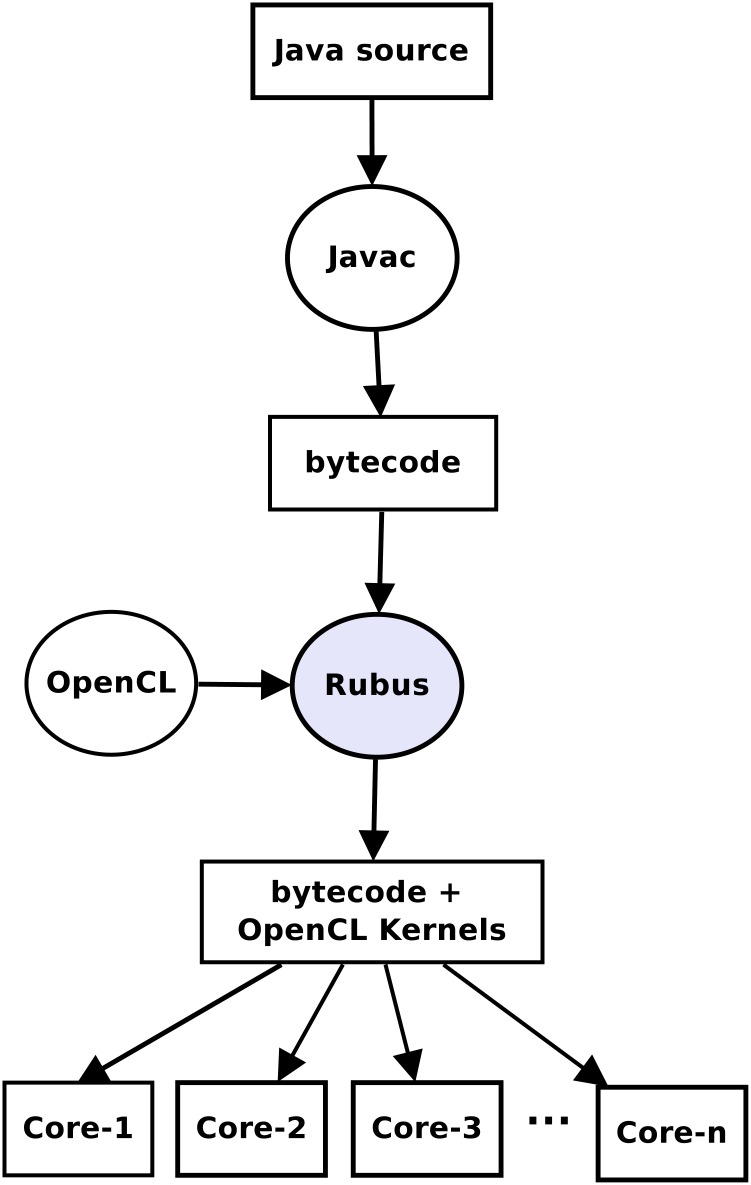
High level overview. High level overview of the transformation of Java code to parallelized code using Rubus.

This section explains how Rubus works and describes the various stages of its code optimization algorithm. Rubus takes Java bytecode as input and outputs modified bytecode, which is ready to run on multiple cores. To this end, Rubus generates OpenCL kernel for each part of code that it deems appropriate for execution on multiple cores. An OpenCL kernel refers to a function executed on different cores with distributed data. The high level stages of Java code transformation using Rubus are shown in Fig 1.

**Fig 2:**
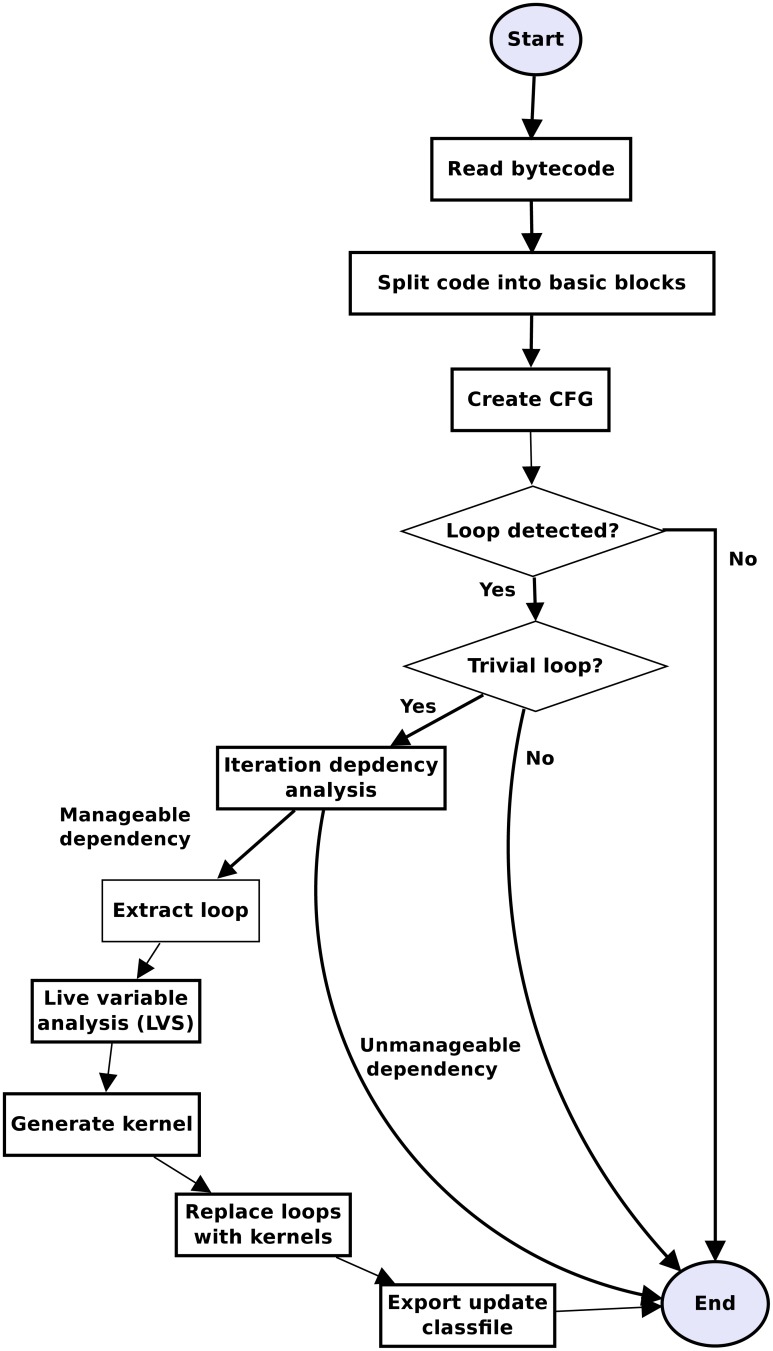
Implementation overview. Implementation overview of Rubus with different stages of the code transformation.

Rubus makes all decisions about code optimization at compile time without taking into consideration the underlying hardware. However, the decision about the data distribution among different cores for parallel execution is carried out at runtime based on the capabilities of the underlying hardware. Fig 2 elaborates the working of Rubus by listing different stages of compilation process. First of all, the bytecode is read by the compiler and divided into basic blocks. Then a Control Flow Graph (CFG) is generated from these basic blocks. The dominator based technique is applied on the CFG to find loop(s). When a loop is found, it is passed to both automated and manual dependency analyzers. In case an analyzer establishes that a loop can be parallelized, it is checked for trivialization. If it is a trivial loop, it is imported into the next phase where live variable analysis is applied on it to find out the arguments for kernel and then kernel is generated in OpenCL based on the body of the loop. After the kernel has been generated a launcher method is also generated in JavaCL to launch this kernel. In the next phase, this loop is replaced with a function call to the launcher method with appropriate arguments. Then the code segments for the kernel and launcher are merged into class file using Javassist library and a new class file is exported. This new class file performs the exact task as the original class file, however, it utilizes multiple GPU/CPU cores to run code in parallel efficiently. We used the vector multiplication code example of Listing 1 to illustrate the impact of different transformation steps.

**public float** [ ] mulVector(**float** [ ] inA, **float** [ ] inB) {

 **int** n = inA.length;

 **float** [ ] result = **new float** [n];

 **for** (**int** i = 0; i < n; i++) {

  result [i] = inA [i] * inB [i];

 }

 **return** result;

}

**Listing 1.** Vector Multiplication Source Code

### 2.1 Reading bytecode

To read and modify bytecode, a bytecode engineering framework called ASM (v3.2) [9] is used. ASM is a Java bytecode manipulation and analysis framework, which uses visitor design pattern to go through the bytecode. Listing 2 has the bytecode generated after the compilation of Java program shown in Listing 1. This bytecode will be used as an example throughout this paper, to illustrate different stages of code transformation using Rubus.

public static float [ ] multiplyVector(float [ ] inA, float [ ] inB) {

 /* L24 */

 0 aload_0;      /* inA */

 1 arraylength;

 2 istore_2;     /* n */

 /* L25 */

 3 iload 2;     /* n */

 4 newarray 6;   /* new float [ ] */

 6 astore_3;    /* result */

 /* L26 */

 7 iconst_0;

 8 istore 4;    /* i */

 10 goto 19;

 /* L27 */

 13 aload_3;    /* result */

 14 iload 4;    /* i */

 16 aload_0;    /* inA */

 17 iload 4;    /* i */

 19 faload;

 20 aload_1;    /* inB */

 21 iload 4;    /* i */

 23 faload;

 24 fmul;

 25 fastore;

 /* L26 */

 26 iinc 4 1;    /* i++ */

 29 iload 4;    /* i */

 31 iload_2;    /* n */

 32 if_icmplt -19;

 /* L29 */

 35 aload_3;    /* result */

 36 areturn;

**Listing 2.** Bytecode of the vector multiplication example whose source code is given in Listing 1.

### 2.2 Basic block

The bytecode in the class file is partitioned into basis blocks. The flow of control can only enter the basic block through the first instruction, which is also called an entry point or leader of a basic block. The flow of control can leave the block only after the execution of its last instruction, called an exit point. There can be no Jump/Halt instruction in between the entry and exit points of a basic block. In other words, a basic block is set of consecutive instructions where only the first instruction of the block can be a target of a jump instruction and only the last instruction can be a jump instruction.

**Fig 3:**
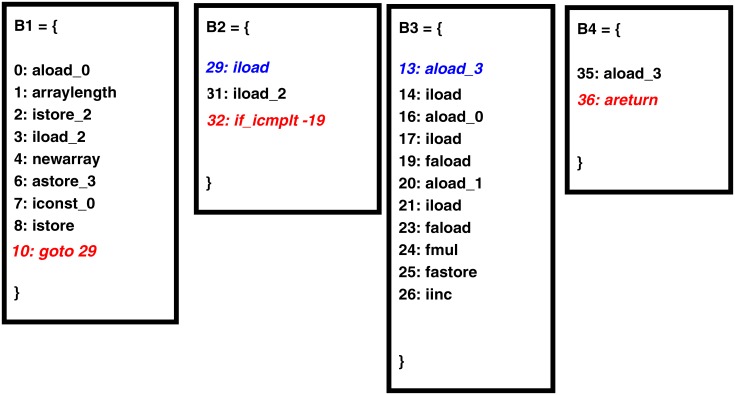
Basic blocks derived from bytecode of Listing 2. The flow of control can only enter the basic block through the first instruction, which is also called an entry point or leader of a basic block.

To find basic blocks, the code analyzer traverses the bytecode and marks a cut wherever it finds an instruction, which transfers or receives the control to/from any other point of the code [10]. This instruction might be a conditional jump, goto or a branch target instruction. After that, the bytecode is split into blocks from these cuts. Applying the above basic block algorithm on the bytecode of the vector multiplication code (Fig 1) produces the basic blocks shown in Fig 3.

In Fig 3, the first block starts with the first statement of the program and ends at line 10 due to a goto statement. This goto statement transfers the program control to line 29. Therefore, block 2 starts at line 29 and ends at line 32 due to an if_icmplt statement. The if_icmplt statement transfers control to line 13 in case the condition is true. Thus, block 3 starts from line 13. The block 3 ends at line 26 because the instruction at line 29 is already the entry point of block 2. if_icmplt transfers control to line 35 in case the condition is false. Therefore, block 4 starts at line 35 and ends at line 36, which is the last statement of the function. In the Fig 3 each jump instruction is assigned red color, whereas a target of jump instruction is colored blue.

### 2.3 Deriving control flow graph

**Fig 4:**
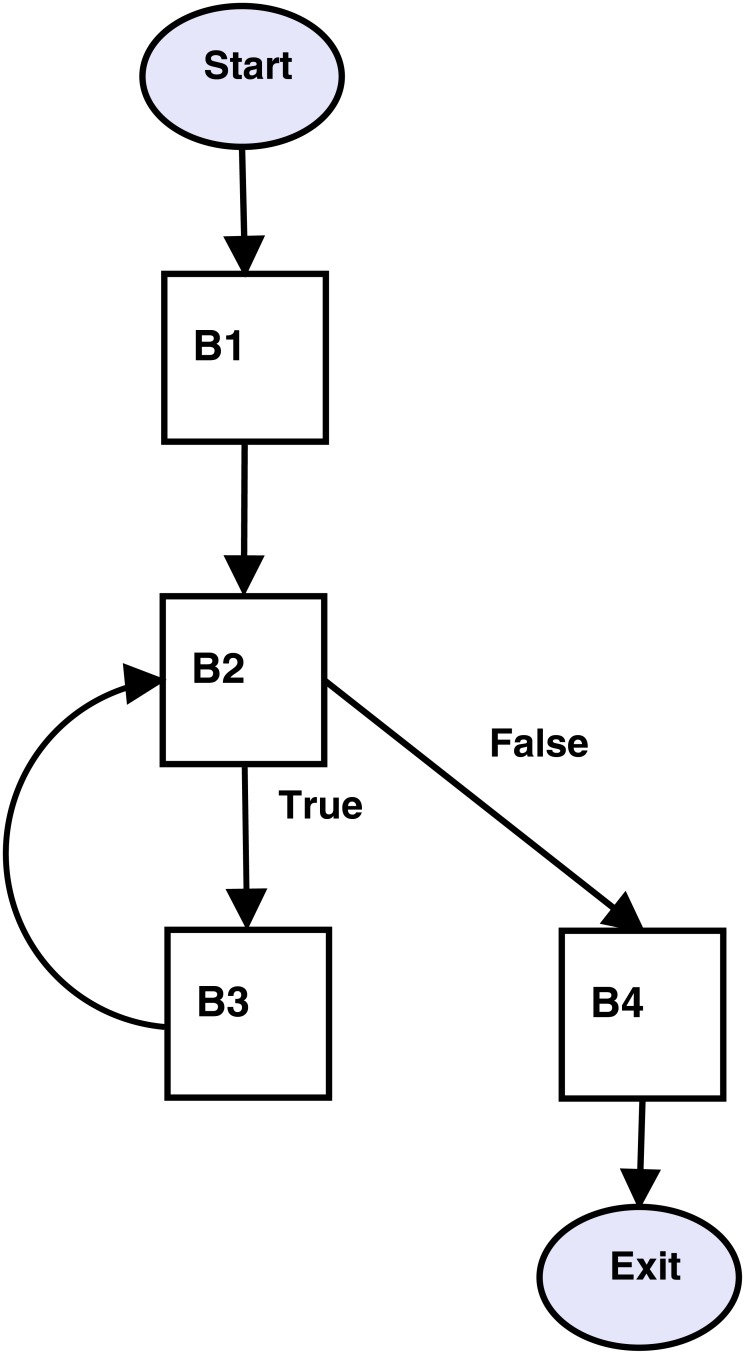
Control flow graph derived from the basic blocks of Fig 3. A control flow graph contains possible paths that might be traversed during the execution of a computer program. A simple control flow graph is shown in the figure is derived directly from the basic blocks of Fig 3.

The flow between the basic blocks is represented using Control Flow Graph (CFG). CFG is a directed graph in which each node represents a basic block and an edge represents the explicit flow of control between the nodes it connects. There is a directed edge from block-x to block-y if the last statement of block-x is a jump statement to the leader (first statement) of block-y. Applying the CFG algorithm on the vector multiplication’s basic blocks of Fig 3 produces CFG shown in Fig 4.

#### 2.3.1 Finding dominator

**Fig 5:**
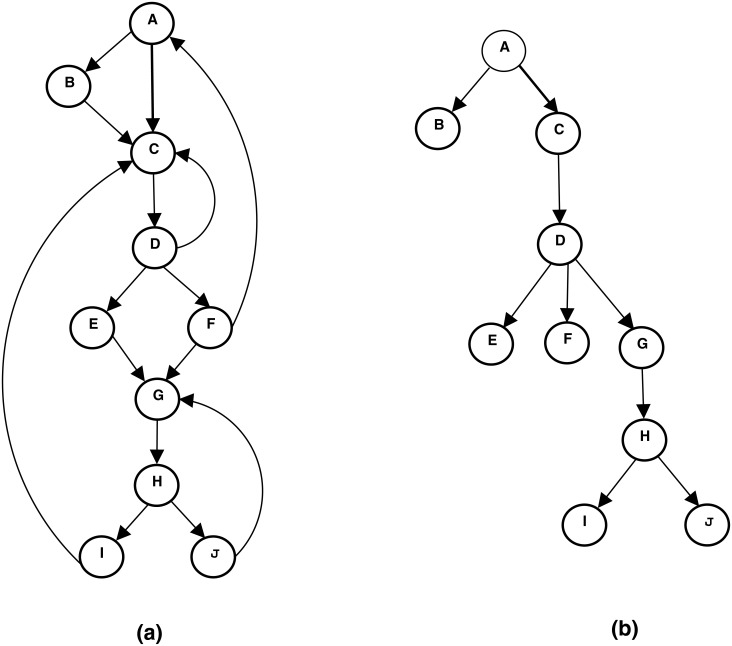
An example of creating the dominator tree (b) from a sample control flow graph (a). A control flow graph contains possible paths that might be traversed during the execution of a computer program. A simple control flow graph is shown in a). The corresponding dominator tree created from the control flow graph is shown in b). The dominator tree is a convenient way to represent the relations of dominator and immediate dominator.

A node *d* dominates a node *n*, if every path of directed edges from the entry node of the CFG to *n* must go through node *d*. By definition, every node dominates itself and the entry node dominates all the nodes in the CFG. A CFG node may have more than one dominator. However, it will always have a unique immediate dominator. The immediate dominator is the *closest* dominator to the CFG node. For example, the nodes J in Fig 5 is dominated by node A, C, D, G and H. However, it has a unique immediate (closest) dominator which is node H.

A dominator tree is a convenient way to represent the relations of dominator and immediate dominator. Dominator Tree contains the entry node as the root node and each node dominates its descendant nodes. Rubus uses a well-known algorithm to find dominators [11] and creates a dominator tree. Fig 5 shows the dominator tree and the corresponding CFG.

### 2.4 Loop detection

A loop in CFG is a set of nodes, say *S*, having a header node *h* such that from every node of *S* there is a path of directed edges leading to *h*. Moreover, nodes in *S* can only be accessed through header node *h* by the nodes not being part of *S*. In order to find loops, Rubus uses a dominator finding algorithm to identify back edges in the CFG as explained below. The presence of a back edge in a graph implies that a graph has a loop. In summary, identifying loops in a CFG is a three step process:

1. Find dominate nodes corresponding to each node of the CFG.
2. Identify all back edges using dominate nodes information.
3. Use the back edges to identify loops.

### 2.5 Natural loop and loop nesting

**Fig 6:**
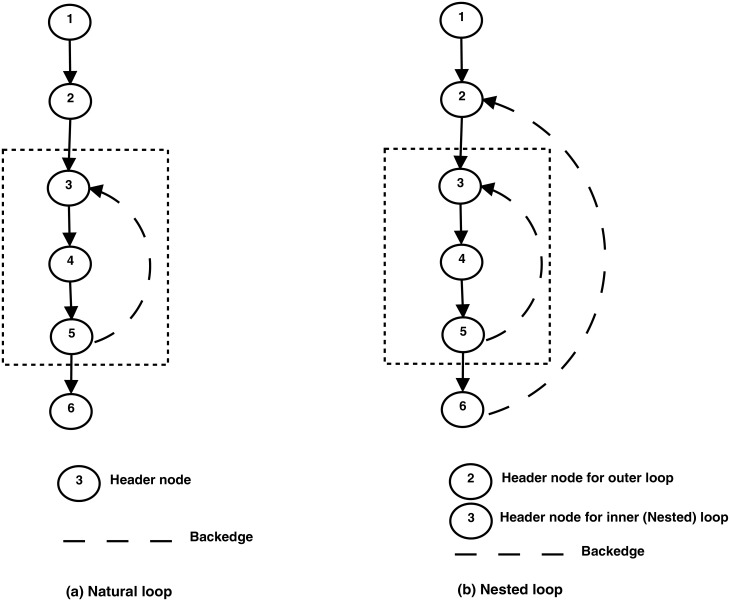
(a) Natural loop (b) Nested loop. (a) A natural loop is a subset of graph *S* having a header node *h* such that every node in subgraph is dominated by *h*. (b) A loop *Y* is the nested or inner loop of loop *X* if *X* and *Y* have different headers *h*_*x*_ and *h*_*y*_.

A natural loop is a subset of graph *S* having a header node *h* such that every node in subgraph is dominated by *h*. There must be a back edge from any node of subgraph to *h*. A back edge is a direct edge from a node *d* to node *h* such that node *d* is dominated by *h*. A loop *Y* is the nested or inner loop of loop *X* if *X* and *Y* have different headers *h*_*x*_ and *h*_*y*_. Moreover, *h*_*y*_ is inside of the body of loop *X*, such that the set of nodes of *Y* are a subset of the nodes of loop *X*. Rubus tries to parallel outer loops first in case of nested loops. Rubus uses the algorithms mentioned in [11], whose basic steps described above determine whether a given loop is trivial or not. Fig 6 shows a natural and a nested loop (in a dotted box) while clearly showing back edges of the CFG.

### 2.6 Finding trivial loop

A natural loop is called a trivial loop if it has only one conditional statement which exits the loop. This condition must be on loop’s incremental indexing variable. The loop limit and dimensions must be known at compile time because we generate separate thread for each index of loop and in multi dimensions for each dimension of the loop and run them on GPU. Incremental variable should increment on a static rate. Rubus accepts trivial loops for further analysis. Rubus uses the well-known algorithms [11], to determine whether a given loop is a trivial loop or not. Running this algorithm on set of loops returns the set of all trivial loops.

### 2.7 Dependency analysis

Rubus provides both automated and manual dependency checks for loops. It does not require programmer input to carryout dependency analyses but a programmer may provide compiler directives to help Rubus to better utilize parallelism. A programmer can direct Rubus to use either automatic or manual dependency analysis, or use both through a command line interface.

**Manual analysis** is based on method annotations provided by the programmer in the Java code. A programmer can specify loop indices by their incremental variable names, which must be considered for parallelization by Rubus. The syntax of method annotation is as shown in Listing 3.

@Transform(loops={“i”})

**public static void** someMethod( ) {

 **for** (**int** i = 0; i < 50; i ++) {

  …

 }

}

**Listing 3.** An Example of Transform Annotation

In contrast, using **automatic analysis**, Rubus finds, analyses and transforms loops by itself. To run a loop in parallel on a GPU, there must not be any RAW (Read-After-Write) and WAW (Write-After-Write) dependencies across iterations. Furthermore, there should not be any loop carried dependencies. Dependency between different iterations of a loop is known as *loop carried dependency*. To ensure that there are no loop carried dependencies, some basic checks are implemented in Rubus. The variables that are local to a loop body never cause the loop carried dependency. Thus dependency checks are only implemented on static/instance variables and the variables defined before the loop body. To ensure that different iterations of loops don’t access the same memory location and a same memory location must not be accessed from any other point of the program, following checks are implemented.

1. Writes are not permitted to fields and arguments. Only local variables are allowed to be written inside the loop body.
2. In case of writing to an array, there must be an increment variable which should be used to access array indices and must be incremented by the same amount in each iteration. The same rule applies if an array is being read and written in the same iteration of the loop. For example, arr[i] = arr[i]*2; is a valid statement but arr[i+1] = arr[i]*2; and arr[i] = arr[i+1]*2; are invalid statements as per restriction.

If a loop does not meet the above mentioned conditions, it would not be accepted for transformation. However, all restrictions and violations are ignored in case of manual analysis.

A programmer configures the use of automatic and manual analyses side-by-side, he can also help Rubus with the *ignore* compiler directive. This directive tells the automated analyzer to not analyze a function which has *ignore* annotated before its body as shown in Listing 4.

@Ignore

**public static void** someMethod( ) {

…

}

**Listing 4.** Ignore Annotation

### 2.8 Loop extraction

A loop is treated as a basic block as well. If this basic block satisfies the imposed conditions, Rubus replaces it with a function call to the kernel launcher and converts this block to OpenCL kernel. All the variables used in loop need to be copied to/from a device memory and passed as arguments to the OpenCL kernel. Rubus uses the live variable analyses technique to identify the arguments for kernel.

### 2.9 Live variable analysis

Live Variable Analysis (LVA) is used to determine if a variable *v* holds a value at point *p* that might be used in the near future. Rubus uses LVA to find out variables needed to copy to the GPU memory before distributing code to that GPU. Furthermore, LVA is also used to find out variables that must be copied back to the host from GPU memory, once GPU completes the execution of a task assigned to it.

### 2.10 Kernel generation in OpenCL

Rubus generates kernel in OpenCL as a string field of the class file being transformed. Rubus also generates a loader function in the class file that defines the size of the workgroup (a.k.a. thread block) and computes the size of the workgroup grid. Subsequently, Rubus sends this information to OpenCL compiler along with kernel code for parallel execution. Based on the underlying hardware specification and information received from Rubus, the OpenCL compiler automatically decides the best possible configuration to distribute threads on various processing units.

The code executed in the kernel saves results in the temporary variables which are copied back, based on live variable analyses, to the host memory at the end of kernel execution.

### 2.11 Kernel launcher generation

The kernel cannot be called directly from Java code. Thus, a Java binding of OpenCL named JavaCL is used to transfer data to GPU, execute kernel and copy result back to the host. JavaCL is used to perform the prerequisite work and for calling kernel using the Java syntax. The prerequisite work includes context creation, device selection, copying data from the host to GPU, loading kernel, executing kernel and copying result back from GPU. JavaCL is chosen in order to avoid C/C++ compiler compatibility issues on different platforms.

### 2.12 Kernel merging

After generating the OpenCL kernel and its launcher (i.e., executor method), the next step is to merge them into the bytecode. For this purpose, a library named Javassist is used, which can compile source code on the fly and merge it into the bytecode.

The kernel is added in the same source file as a string field having the same name as kernel. The kernel executor code is added as a function in the same class file having the same name and parameter as kernel. The modified class file is exported to a given destination.

## 3 Performance evaluation

We used several algorithms and benchmarks from different areas of life to evaluate Rubus. This section compares the performance of Rubus with benchmarks written using Java as well as with programs developed using a parallel API from AMD, called Aparapi [7]. All of Aparapi’s benchmarks are taken from Aparapi’s code repository, which are handwritten and optimized by AMD developers themselves, so that an unbiased and neutral comparison with Rubus could be drawn. Aparapi provides a high-level Java API to write code for a GPU. Aparapi decides at *runtime* whether it should convert the code into OpenCL or run the code using Java threads. In contrast, Rubus transforms the code into OpenCL using decisions made at compile time. Making decisions at runtime has overhead that sometimes adversely affects the performance of programs written using Aparapi, making them run slower as compared to Rubus.

### 3.1 Experiments setup and hardware specifications

Junitbenchmark is a tracker program that can be used to track time and analyze performance of Java programs [12]. To evaluate the performance of Rubus on several benchmarks and generate results, we have employed Junitbenchmark, with some modifications. These modifications are carried out to make charts and results more informative.

To produce results of each benchmark, 10 JVM warm-ups, followed by 10 benchmark rounds were executed for a given input size. The JVM warm-up refers to the time it takes for the JVM to find the hotspots of the code hence warm-up results are ignored. Instead, the subsequent 10 benchmark rounds average is used as the final result. All benchmarks are evaluated on HP Pavilion DV4 machine which has following specifications.

**CPU:** Inter(R) Core(TM) i7-2670QM @ 2.20GHZ 2.20GHZ

**GPU:** GeForce GT 630M 96 Cores 800 MHz—Texture Fill Rate 12.8 billion/sec

**GPU Memory:** DDR3/GDDR5, 2GB, Interface Width 128bit, Memory Bandwidth 32GB/sec

**OpenCL:** 1.1 Supported

**OpenGL:** 4.4 Supported

**RAM:** 4.00 GB

**Operating System:** Microsoft Windows 8.1—64 bit

The following set of scientific problems and algorithms are chosen to evaluate the performance of Rubus.

### 3.2 Matrix multiplication

Matrix multiplication is a widely used mathematical operation in graphics processing and other scientific computations. For example, matrix multiplication can be used to solve recurrence relations and to represent linear transformations between vector spaces. We used *n* × *n* matrices with values of *n* as 32, 64, 128, 256, 512, 1024, and 2048 to produce results.

**Fig 7:**
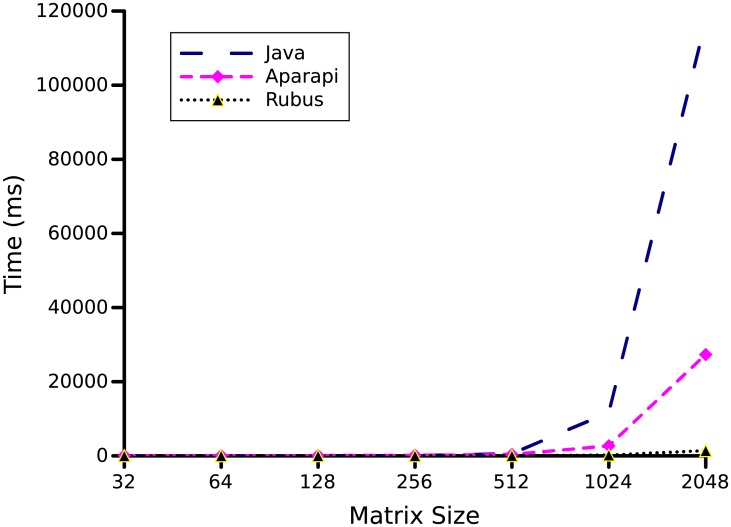
Matrix multiplication benchmark. *n* × *n* matrices with values of *n* as 32, 64, 128, 256, 512, 1024, and 2048 is used to produce results.

The graph in Fig 7, shows the performance comparison of Java, Rubus and Aparapi code for matrices of different sizes. On a small input size, Rubus and Aparapi took more execution time than the original Java code (not noticeable in graph of Fig 7). This is because, on small input size the overhead of data transfer to different cores exceeds the benefit of speedup. However, as the size of input increases, the benefit of parallelism become more visible as both Rubus and Aparapi require significantly less time than sequential programs written in Java. Furthermore, on large input sizes, Rubus’s performance is many times better than Aparapi. That is, a speedup of up to 83.93 times is experienced by the Rubus and only 4.3 times speedup is experienced by Aparapi as compared to Java code.

### 3.3 Convolution

**Fig 8:**
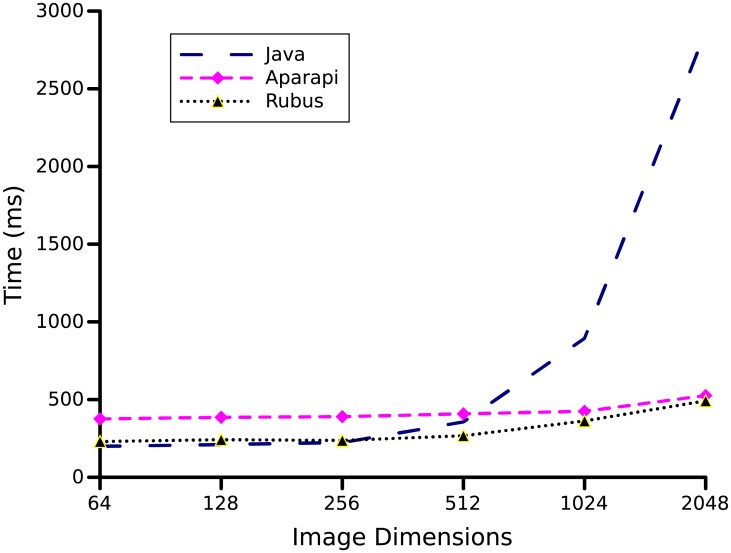
Convolution benchmark. Convolution filtering is used to modify the characteristics of an image. The images of dimensions *n* × *n*, with *n* as 32, 64, 128, 256, 512, 1024, 2048, and 4096 are used to produce above graph.

Convolution filtering is used to modify the characteristics of an image, that is widely used in the field of image processing. Convolution function takes image and a matrix of number as input. The matrix is used to filter the input image and to produce a transformed image as an output. Convolution is often used to smooth, sharpen, enhance or blur an image. We used images of dimensions *n* × *n*, with *n* as 32, 64, 128, 256, 512, 1024, 2048, and 4096. As shown in Fig 8 performance of Rubus and Aparapi is almost same on small input sizes. Whereas, Rubus performs slightly better (5.83x) than Aparapi (5.45) on image of size 2048 × 2048.

### 3.4 Mandelbrot set

**Fig 9:**
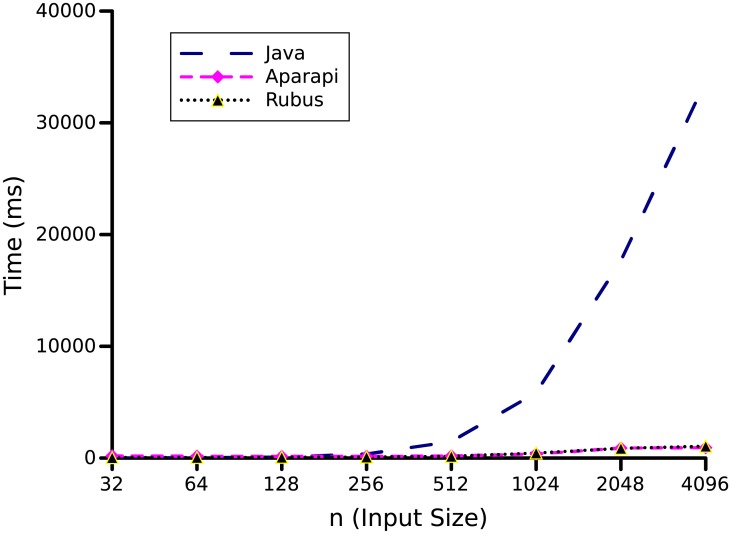
Mandelbrot benchmark. The Mandelbrot set is the problem of complex dynamics. Data set size, *n*, with values 32, 64, 128, 256, 512, 1024, 2048, and 4096 is used to evaluate Rubus.

The Mandelbrot set is the problem of complex dynamics, which was introduced and investigated by French mathematicians Pierre Fatou and Gaston Julia [13]. It is the set of complex numbers, which is obtained from the equation $z_{\left(n+1\right)}=z_{n}^{2}+c,z_{0}=c$. Here *c* is a complex number. To produce images for a Mandelbrot set, the real and imaginary parts of each number are treated as image coordinates. Pixels are colored according to the speed the sequence changes. We used data set size, *n*, with values 32, 64, 128, 256, 512, 1024, 2048, and 4096 to evaluate Rubus. As shown in Fig 9, Aparapi performance is bit better than the Rubus when used to generate Mandelbrot set. A speedup of 31.76x is experienced on input size 4096, which is less than the Aparapi which is 36.2x. Recall that the Aparapi code is manually written and carefully optimized for parallelism as compared to the automatically generated code by Rubus. Thus, for this problem Rubus underperformed slightly as compared to Aparapi.

### 3.5 N-body simulation

**Fig 10:**
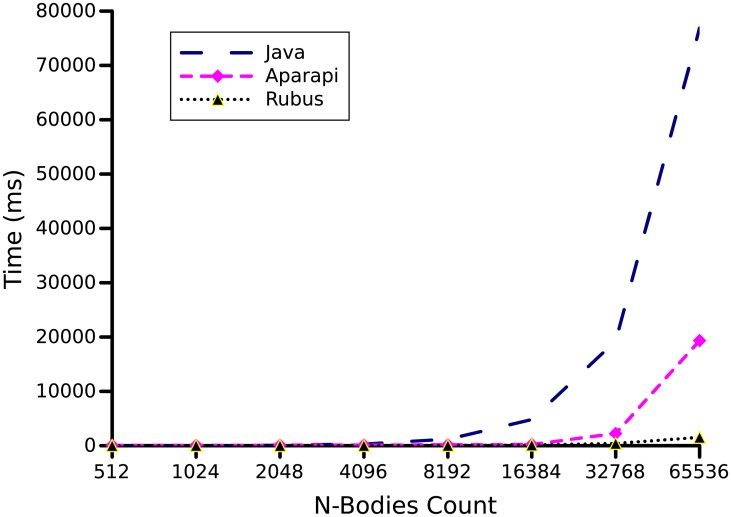
N-body benchmark. N-body Simulation is a well-known problem in astronomy and physics. The number of bodies as 256, 512, 1024, 2048, 4096, 8192, 16384, 32768, and 65536 are used to produce above graph.

N-body Simulation is a well-known problem in astronomy and physics, that is simulated on a system of particles. The particles are influenced by physical forces. N-body simulation computes new positions of particles influenced by some physical forces like gravity. We used the number of bodies as 256, 512, 1024, 2048, 4096, 8192, 16384, 32768, and 65536. As shown in Fig 10, a speedup of 50*x* was achieved by the Rubus on input size 65536 while on the same input Aparapi provides only 21.39*x* speedup as compared to its Java implementation.

### 3.6 Squares

**Fig 11:**
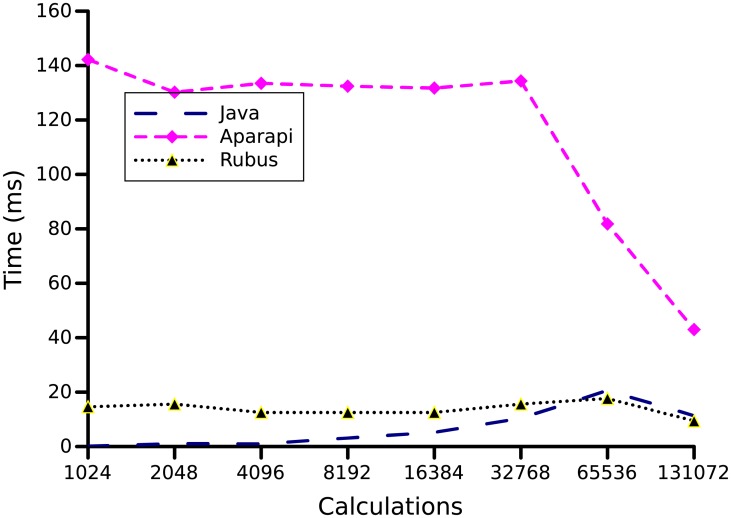
Squares benchmark. In this benchmark, squares of values of a given vector are computed. The array sizes of 1024, 2048, 4096, 8192, 16384, 32768, 65536, and 131072 are used to produce above graph.

In this benchmark, squares of values of a given vector are computed. This benchmark uses the Math.pow() function. We used array sizes of 1024, 2048, 4096, 8192, 16384, 32768, 65536, and 131072. As shown in Fig 11, on large input, Rubus provided a speedup of 1.21x. The reason for this small speed up is that the problem is data driven with small computation therefore the kernel contains small computation and the computational gain in parallel version of Rubus is subsided by the large data transfer overhead. In contrast, Aparapi is slower by 3.8x as Aparapi performance decrease while using the math functions.

Another important factor is GPU’s memory which can affect the execution speed of a program. If GPU’s memory is smaller than the data size, it may take multiple iterations to copy the required data to GPU’s memory before execution. This means that the data copy overhead would be higher, which would result in speed down of the transformed code. Increasing data size would also decrease the performance accordingly.

### 3.7 Real-time movie convolution

In the previous sections we have provided results on five different benchmarks. Each of these benchmarks has numerous applications, from solving mathematical problems to computer vision. This subsection presents one such application by applying convolution in real-time on a well-known high quality Big Buck Bunny’s video [14]. We have developed a tool to apply convolution on different videos. Our tool takes as input a video and a set of filter parameters to be applied on that video. Based on the input it shows the live effects of the filter parameters being applied on the video while showing the performance ratios of Rubus and Aparapi with respect to Java.

**Fig 12:**
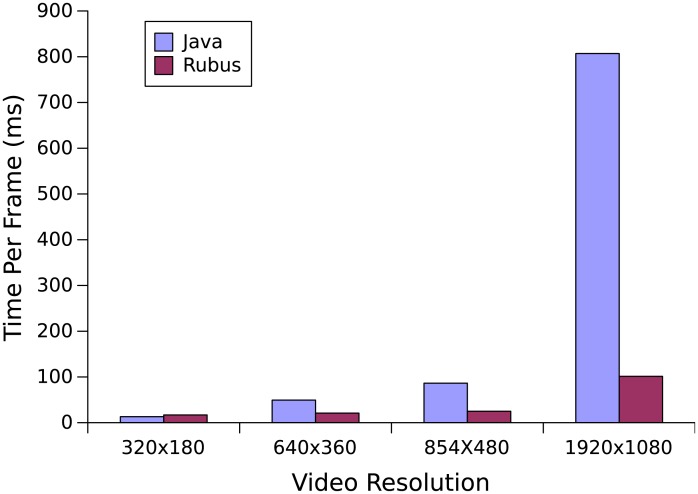
Real-time video convolution. The above graph is produced by applying convolution in real-time on a well-known high quality Big Buck Bunny’s video [14]. Different 3*x*3 matrices are used to perform various filters on the video.

Different 3*x*3 matrices are used to perform various filters on the video including edge detection, sharpen, box blur, Gaussian blur, Emboss, and outline detection. Based on these convolutions, an average transformation time has been taken over four different video resolutions. Fig 12 shows that with the increase in the video resolution, Rubus performance becomes significantly better than serial Java program.

Finally, Table 1 shows the summary of our benchmarking. It shows the speedups achieved by using Rubus and Aparapi with respect to Java code. Java execution time is normalized to 1 and performance of Rubus and Aparapi is calculated accordingly.

**Table 1:** Summary of results: Performance ratio of Rubus and Aparapi versus Java.

Benchmarks	Rubus	Aparapi
Matrix Multiplication	83.93	4.3
Convolution	5.83	5.45
Mandelbrot	31.76	36.2
N-Body Simulation	50	21.39
Squares	1.21	−3.8

## 4 Related work

In this section, we have described various tools that can utilize the computational power of multiple processing units available in the modern computers. To this end, we have divided these tools in two different main categories that are further divided into some subcategories. Firstly, we have described the tools that operate on Java source or bytecode. As these tools are somewhat similar to Rubus hence we have also highlighted Rubus superiority over them. Secondly, we have listed notable tools that work with languages other than Java or Java bytecode.

### 4.1 Java tools for parallelism

Many existing tools work on the Java source code such as, JavaCL [6], JCUDA [4], JOCL [5], Rootbeer [8], Aparapi [7] and HJ-OpenCL [15]. Unlike these tools, Rubus works on Java bytecode. Thus, Rubus can parallelize code for all those programming languages that produce intermediate Java bytecode. Furthermore, The aforementioned solutions are different from Rubus in many ways. JavaCL and JOCL are Java bindings of OpenCL, which provides the facilities to use OpenCL/CUDA in Java syntax. In contrast, Rubus is a compiler which seamlessly performs optimizations in the Java bytecode to take advantage of the underlying GPUs. Rootbeer and Aparapi are user-friendly libraries which provide a very high-level interface to work with CUDA [3] and OpenCL [16] respectively. However, in order to use them the user needs to know some GPU programming concepts and should be well aware of the art of parallel programming. In contrast, no knowledge of GPU and parallel programming is needed to make code parallel using Rubus.

HJ-OpenCL [15] is also a compiler, which transforms Habanero-Java (HJ) code into OpenCL. HJ is an extension for Java, that provides some additional constructs for parallel programming using multicore processors. Unlike Rubus, HJ-OpenCL works with HJ only, which requires the use of a specific HJ compiler to compile the code. Another main difference between Rubus and HJ-OpenCL is that the HJ-OpenCL uses a parallel construct to manually identify the code snippets that can run in parallel. On the other hand, Rubus does not need any such type of hints from the programmer to transform the code for parallelism.

Java-gpu [17] on which Rubus is based performs similar optimization on the bytecode to make it run in parallel. However, it compiles the code into CUDA code and supports Nvidia’s GPU only. On the other hand, Rubus supports all OpenCL-enabled GPUs and CPUs, and requires no extra tools to be installed except OpenCL drivers.

### 4.2 Non-Java tools for parallelism

Many tools are available that can be used to transform programs written in languages other than Java into OpenCL or CUDA. We briefly discuss some of these tools in this section.

#### Python

For Python there exist PyCuda [18], PyOpenCL [19], and CopperHead [20]. PyCUDA [18] is an API written in Python which lets user work with CUDA using Python. It requires the kernel to be written in CUDA code. PyOpenCL [19] is a Python binding of OpenCL to work with parallel hardware. CopperHead [20] is another framework written in Python to work with Nvidia’s GPU and multicore CPUs using OpenMP.

#### JavaScript

RiverTrail [21] extends JavaScript and adds some parallel constructs which are then converted to OpenCL at run time. WebCL [22] is a JavaScript binding of OpenCL which runs in a browser without requiring any browser plugin.

#### C/C++

Some other tools transform C/C++ code for parallelism. These tools include ArBB [23], OpenVIDIA [24], and OpenACC [25]. Intel ArBB [23] is a C++ library which works with standard C++ compilers. It focuses on thread parallelism by executing code on multi-core systems. OpenVIDIA [24] is an API written in C/C# to perform computer vision and image processing operations on GPUs. OpenACC [25] is a programming standard for parallel computing. Using OpenACC a user can specify C, C++ and Fortran code portions using some directives to run them in parallel on parallel hardware.

#### Others

Firepile [26] is a library for Scala developers to work with GPUs. Code is completely written in Scala and Firepile converts kernel code into OpenCL to run it on a GPU. ASDP [27] is a domain specific language based on ActionScript designed to use GPU and multicore CPU to achieve data level parallelism.

## 5 Conclusion

This paper presents the design and implementation of a new compiler Rubus for seamless parallelism. The Rubus compiler takes as input serial code, analyses and transforms it into parallel code so that it can be executed on multiple cores of CPUs/GPUs. Although the process of transformation is automated but the programmer may optionally provide *hints* to the compiler to further improve the compiler’s performance. The Rubus is designed on principle that significant data level parallelism can be achieved by executing concurrent loop iterations in parallel. Rubus transforms the loops into OpenCL kernel and merges generated code with actual bytecode. Main advantage of the Rubus is that the original source code remains simple, robust and easy to debug while the intermediate code generated by Rubus is prime to run in parallel. Furthermore, a legacy sequential program or a library that was written years before the invention of GPU may also run in parallel using the Rubus compiler. Rubus performance on vast varieties of programs from different areas of life has been evaluated and a significant execution speedup has been experienced on large input sizes. Compared to Java implementation, execution speedup up to 84 times has been achieved by Rubus on a basic GPU having only 96 cores.

## 6 Limitations and future work

In this section we highlight the limitations of Rubus and suggest possible future work. Many of these limitations are also mentioned in other relevant sections but this section consolidates them in one place. A majority of the existing tools heavily rely on programmer’s input to produce parallel code. In contrast, Rubus uses data-flow analysis to find, analyze and transform loops automatically. To run a loop in parallel on a GPU, there must not be any RAW (Read After Write) and WAW (Write After Write) dependencies across iterations. Furthermore, there should not be any loop carried dependencies. The current data-flow analyzer of Rubus is not inter-procedural. Thus it cannot detect if two methods are data independent from each other and can be executed in parallel. The data-flow analyzer of Rubus also cannot detect if two Java threads may be executed in parallel. To compensate for some of these limitations Rubus allows programmers to use compilers directives to improve it’s capabilities.

Planned future works include the support of multiple if/else statements inside the loop body, custom data types, string, multi-dimensional arrays and collection. To improve transformation, some advanced data dependency analysis techniques like GCDTest [28] will be implemented. Rubus currently copies all data to the GPU’s global memory, which takes more time to access as compared to local and the shared memory of each core and results in slowdown of the execution speed. In future, local and shared memory block of GPU would be used where possible to further increase the performance. Furthermore, Rubus copies the whole data arrays to the GPU, incurring overhead. We will optimized Rubus, in future, to identify and copy the exact indexes of array being used for additional speedup.

Current release of Rubus uses JavaGPU to parse classfiles thus Rubus does not work on all the classfiles that cannot be correctly parsed using JavaGPU. To fix that issue, in a future release, we will write our own classfile parser.

## Supporting information

S1 Table Java maths functions’ support and mapping in OpenCL. (PDF)

S1 Fig Graphical user interface for Rubus compiler. (EPS)

S2 Fig The performance ratios of Rubus and Aparapi versus Java. (EPS)

S1 File Pseudocode of various algorithms used by Rubus. (PDF)

S2 File Source code transformation using Rubus. (PDF)

S3 File How to use Rubus. (PDF)
