# Supplementary material for: Rubus: A compiler for seamless and extensible parallelism
Source: PLoS One. 2017 Dec 6;12(12):e0188721. doi: 10.1371/journal.pone.0188721 (PMC5718508; doi:10.1371/journal.pone.0188721)
Supplement: S1 Table — (PDF) [file pone.0188721.s001.pdf]

Java maths function support and mapping in OpenCL shown in Table 1

| **Java** | **OpenCL** | **Java** | **OpenCL** |
|---|---|---|---|
| abs (float, double) | fabs | abs | abs |
| ceil | ceil | floor | floor |
| rint | rint | round | round |
| tan | tan | min (float, double) | fmin |
| min | min | acos | acos |
| max (float, double) | fmax | max | max |
| sqrt | sqrt | exp | exp |
| log | log | cos | cos |
| pow | pow | sin | sin |
| atan | atan | cbrt | cbrt |
| copysign | copysign | cosh | cosh |
| expm1 | expm1 | log10 | log10 |
| hypot | hypot | log1p | log1p |
| nextafter | nextafter | asin | asin |
| atan2 | atan2 | sinh | sinh |
| tanh | tanh | | |

Table 1: Supported Math Functions
