# Supplementary material for: Rubus: A compiler for seamless and extensible parallelism
Source: PLoS One. 2017 Dec 6;12(12):e0188721. doi: 10.1371/journal.pone.0188721 (PMC5718508; doi:10.1371/journal.pone.0188721)
Supplement: S1 File — (PDF) [file pone.0188721.s004.pdf]

Pseudocode of various algorithms used by Rubus

July 25, 2017

1 Basic Block

Input: Set of statements where S_i denotes the i_{th} statement of a program.
Output: Set of all leaders and set of basic blocks where block(j) contains all statements having leader j.

```
n = |statements|
// first node of a program is by default a leader
leaders = {1}
for i = 1 to n
 if S_i is a jump, then
   leaders = leaders ∪ targets where jump can pass the control ∪ instruction
    immediately followed by the jump statement
end for
leaderList = leaders
while leaderList ≠ empty do
        j = remove a statement from leaderList
        // leader statement is first statement of corresponding block
        block(j) = {j}

        for i = j + 1 to n  and i ∉ leaders
                block(j) = block(j) ∪ {i}
        end for
end while
```

Listing 1: Basic Blocks Algorithm

2 Deriving Control Flow Graph

Input: Set of basic blocks.
Output: Control Flow Graph.

```
n = |block|
for i = 1 to n
   x = last statement of block i
   if x is a jump, then
     for each target block y of x
       add edge from block i to block y
     end for
   end if
   if x is conditional jump then
     add edge from block i to block i+1
   end if
end for
```

Listing 2: Control Flow Graph Algorithm

3 Finding Dominator

Input: Flow graph G having set of nodes N, set of Edges E and starting node s.
Output: Dominator set dom, where dom[n] denotes the set of nodes that dominate node n.

```
dom[s] = {s}
for i ∈ N − { s } do
 dom[i] = N
end for
while any dom[i] changes, do
        for j in N − { s }  do
           dom[j] ={ j } ∪ { ∩ p ∈ predecessor [j]dom[p] }
        end for
end while
```

Listing 3: Finding Dominator Algorithm

4 Dominator Tree

Input: Dominator set dom where dom[n] denotes the set of nodes that dominate node n and start node s
Output: Dominator Tree

```
T = Empty Tree
T.root = s
for each unvisited node d in T
        mark d visited
        for each node n in dom set
                if dom[n] contains d
                        remove d from dom[n]
                        if dom[n] contains only one node(n)
                                make n the child of d
                        end if
                end if
        end for
end for
```

Listing 4: Dominator Tree Algorithm

5 Natural Loop

Input: Dominator tree T
Output: Set of loops

```
for each node h in T
 for each node n in T having back edge from n to h
  define the loop L with header h and back edge from n to h
```

```
  Mark h as visited such that search of nodes must not add it twice
  Start back depth first search from n for h and add all visited nodes in loop L
  end for
end for
```

Listing 5: Loop Finding Algorithm

6 Loop Nesting

Input: Set of loops L, set of all loop headers H
Output: Set of nested loops

```
for each node h in H
   for each loop l in L
     for each node h1 in L
       if h == h1
        make h a child of h1
        remove h from H
        end if
     end for
   end for
end for
```

Listing 6: Loop Nesting Algorithm

7 Trivial Loop

Input: Set of loops L
Output: Set of Trivial loops T

```
for each loop l in L, do
 let bList is the list of loop branches
 if count of bList > 1
    continue
  end if
  if branch statement of loop is not a conditional branch i.e. a break statement
    continue
  end if

  if condition is not comparison of increment variable
    continue
  end if

 T = T ∪ l

end for
```

Listing 7: Loop Trivializing Algorithm

8 Live Variable Analysis

Input: A flow graph with def and use computed
Output: Set of live variables before and after all blocks, in[B] and out[B] For each block in flow graph except exit

```
in[B] = Empty;
while any in[X] updated
   for each basic block B except Exit
      in[B] = ∪ in[S], for all successor S of B
      out[B] = use[B] ∪ (out[B] − def[B])
   end for
end while
```

Listing 8: Live Variable Analysis Algorithm

9 Kernel Generation in OpenCL

Algorithm

Input: A set of Basic Blocks K to export as kernel
Output: OpenCL kernel as a string

```
kernelStr = kernel prefix
for each block b in K
  for each instruction i in b
    kernelStr = kernelStr ∪ i converted to OpenCL C
```

Listing 9: Kernel Transformation Algorithm

10 Kernel Launcher Generation

Algorithm

Input: Argument list and kernel string
Output: Kernel launcher code

```
argumentList = List of arguments for kernel using Live Variable Analysis
Define function with argumentList
Get OpenCL context
Create properties queue
Create kernel from string
Copy data from host to device
Set kernel arguments
Set kernel thread dimensions
Execute the kernel
Copy result back from device
```

Listing 10: Kernel Launcher Generation Algorithm
