# Supplementary material for: Rubus: A compiler for seamless and extensible parallelism
Source: PLoS One. 2017 Dec 6;12(12):e0188721. doi: 10.1371/journal.pone.0188721 (PMC5718508; doi:10.1371/journal.pone.0188721)
Supplement: S2 File — (PDF) [file pone.0188721.s005.pdf]

1 Source code transformation using Rubus

1.1 Source code:

```
public class VectorMultiplication {
        public static void main(String[] args) {
                int n = 128;
                float inA [] = new float[n];
                float inB [] = new float[n];
                // assign random values..
                for (int index = 0; index < inA.length; index++) {
                        inA[index] = (float)Math.random()*100;
                        inB[index] = (float)Math.random()*100;;
                }
                float result [] = multiplyVector(inA, inB);
                for (int index = 0; index < result.length; index++) {
                        System.out.println(result[index]);
                }
        }
        @Transform(loops={"i"})
        public static float[] multiplyVector(float[] inA, float[] inB) {
                int n = inA.length;
                float[] result = new float[n];
                for (int i = 0; i < n; i++) {
                                result[i] = inA[i]*inB[i];
                }
                return result;
        }

}
```

Listing 1: Vector Multiplication Complete Source Code

1.2 Transformed code using Rubus:

```
public class VectorMultiplication
{
  private static final String kernel_1856089607 = "
      __kernel void kernel_1856089607
        ( const int limit0, __global float* v3_2891
            , __global float* v0_2891,
        int v4_INT, __global float* v1_2891)
        {
                int dim0 = get_global_id(0);
                v4_INT += 1 * dim0;
```

```
                    if(v4_INT >= limit0) return;
                    {
                            __global float* t0 =
                               v3_2891;
                            int t1 = v4_INT;
                            __global float* t2 =
                               v0_2891;
                            int t3 = v4_INT;
                            float t4 = t2[t3];
                            __global float* t5 =
                               v1_2891;
                            int t6 = v4_INT;
                            float t7 = t5[t6];
                            float t8 = t4*t7;
                            t0[t1] = t8;
                    }

                    {
                            v4_INT += 1;
                    }

            }";

                }";

private static CLContext context = JavaCL.
   createBestContext();

public static void main(String[] paramArrayOfString)
{
  int i = 128;
  float[] arrayOfFloat1 = new float[i];
  float[] arrayOfFloat2 = new float[i];

  for (int j = 0; j < arrayOfFloat1.length; j++) {
    arrayOfFloat1[j] = ((float)Math.random() * 100.0F);
    arrayOfFloat2[j] = ((float)Math.random() * 100.0F);
  }
  float[] arrayOfFloat3 = multiplyVector(arrayOfFloat1,
      arrayOfFloat2);
  for (int k = 0; k < arrayOfFloat3.length; k++)
    System.out.println(arrayOfFloat3[k]);
}

public static float[] multiplyVector(float[]
   paramArrayOfFloat1, float[] paramArrayOfFloat2)
```

```
  {
    int i = paramArrayOfFloat1.length;
    float[] arrayOfFloat = new float[i];
    int j = 0; kernel_1856089607(i, arrayOfFloat,
        paramArrayOfFloat1, j, paramArrayOfFloat2);

    return arrayOfFloat;
  }

  public static void kernel_1856089607(int paramInt1,
      float[] paramArrayOfFloat1, float[]
      paramArrayOfFloat2, int paramInt2, float[]
      paramArrayOfFloat3)
  {
    CLDevice.QueueProperties[] arrayOfQueueProperties = {
         CLDevice.QueueProperties.ProfilingEnable };
    CLQueue localCLQueue = context.createDefaultQueue(
        arrayOfQueueProperties);
    String[] arrayOfString = { kernel_1856089607 };
    CLKernel[] arrayOfCLKernel = context.createProgram(
        arrayOfString).createKernels();
    CLKernel localCLKernel = arrayOfCLKernel[0];
    CLBuffer localCLBuffer1 = context.createBuffer(CLMem.
        Usage.InputOutput, Pointer.pointerToFloats(
        paramArrayOfFloat1), true);
    CLBuffer localCLBuffer2 = context.createBuffer(CLMem.
        Usage.Input, Pointer.pointerToFloats(
        paramArrayOfFloat2), true);
    int i = paramInt2;
    CLBuffer localCLBuffer3 = context.createBuffer(CLMem.
        Usage.Input, Pointer.pointerToFloats(
        paramArrayOfFloat3), true);
    localCLKernel.setArgs(new Object[] { paramInt1,
        localCLBuffer1, localCLBuffer2, i, localCLBuffer3
        });
    int j = paramInt1 - paramInt2 / 1;
    CLEvent[] arrayOfCLEvent = { null };
    CLEvent localCLEvent = localCLKernel.enqueueNDRange(
        localCLQueue, new int[] { j }, arrayOfCLEvent);
    localCLQueue.finish();
    localCLBuffer1.read(localCLQueue, new CLEvent[] {
        localCLEvent }).getFloats(paramArrayOfFloat1);
  }
}
```

Listing 2: Vector Multiplication Transformed Code
