# Supplementary material for: Rubus: A compiler for seamless and extensible parallelism
Source: PLoS One. 2017 Dec 6;12(12):e0188721. doi: 10.1371/journal.pone.0188721 (PMC5718508; doi:10.1371/journal.pone.0188721)
Supplement: S3 File — (PDF) [file pone.0188721.s006.pdf]

1 How To Use Rubus

Rubus is distributed as a single Jar in binary form. This Jar contains the Rubus GUI as well. Rubus can be used from command line or with GUI interface. The command line interface has a number of optional arguments. These are shown in the table below:

Option	Description
-auto	If -auto flag is given, Rubus will perform automated analysis.
-manual	If -manual flag is given, Rubus will perform annotation based analysis.
-clean	Destination folder will be deleted before transformation.
-debug	Rubus will show all log messages.
-export	Export generate kernel and executor Java code.
-open	Open generated source file after transformation.
–dist	Destination path to export transformed code.
–path	Class path to serach class files from. All path should be separated by comma ','.

Table 1: Compiler Options

After options, all class and Jar files should be given, seprated by comma. To perform both auto and manual analysis, both -auto and -manual flags should be given. All arguments can be printed to the console using "Rubus.jar -help" command.

---

java Rubus [options...] arguments...
-auto : Should perform auto analysis
-clean : Clear destination dir before this run
-debug : Should show logs
-export : Export generate kernel and executor code
-manual : Should perform manual analysis
-open : Open generated source file
–dist FILE : Destination path to export transformed code
–path FILE : Class path to serach class files from. All path should be separated by comma ','.
Example: java Rubus –dist FILE -auto -clean -debug -export -manual -open -path FILE

If no arguments are given, Rubus will open the following compiler GUI. All options are also available in GUI enviournment.

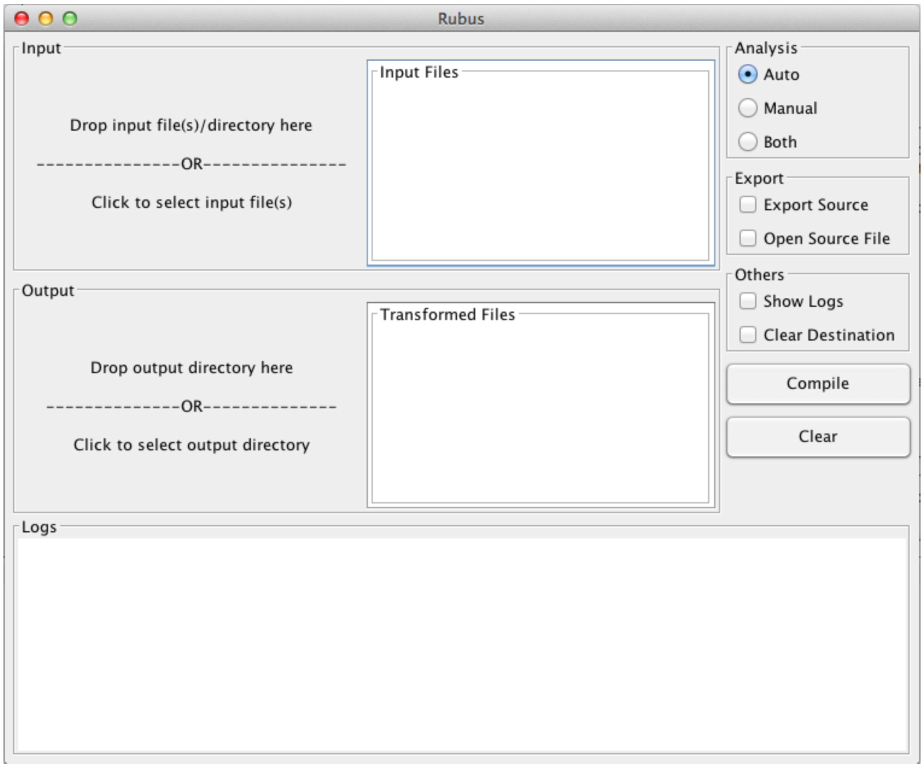

Figure 1: Rubus GUI
